# Supplementary material for: Comparative genomics provides new insights into the diversity, physiology, and sexuality of the only industrially exploited tremellomycete: Phaffia rhodozyma
Source: BMC Genomics. 2016 Nov 9;17:901. doi: 10.1186/s12864-016-3244-7 (PMC5103461; doi:10.1186/s12864-016-3244-7)
Supplement: Additional file 6: — List of orphan genes with links to PFAM (related to Additional file 1: Table S1). (ZIP 1428 kb) [file 12864_2016_3244_MOESM6_ESM.zip › BLAST_HTML_FTR/G01838_P.html]

BLAST Search Results


```
BLASTP 2.2.27+


Reference:
Stephen F. Altschul, Thomas L. Madden, Alejandro A. Schäffer,
Jinghui Zhang, Zheng Zhang, Webb Miller, and David J. Lipman (1997),
"Gapped BLAST and PSI-BLAST: a new generation of protein database
search programs", Nucleic Acids Res. 25:3389-3402.


Reference for
composition-based statistics:
Alejandro A. Schäffer, L. Aravind, Thomas L. Madden, Sergei
Shavirin, John L. Spouge, Yuri I. Wolf, Eugene V. Koonin, and
Stephen F. Altschul (2001), "Improving the accuracy of PSI-BLAST
protein database searches with composition-based statistics and
other refinements", Nucleic Acids Res. 29:2994-3005.


Database: nr
           71,551,133 sequences; 26,053,659,533 total letters


Query= G01838_P

Length=837
                                                                      Score     E
Sequences producing significant alignments:                          (Bits)  Value

emb|CED83616.1|  WW domain [Xanthophyllomyces dendrorhous]            1653    0.0  
ref|XP_001884802.1|  predicted protein [Laccaria bicolor S238N-H8...  47.0    0.11 
ref|XP_001838984.2|  hypothetical protein CC1G_05537 [Coprinopsis...  45.4    0.29 
ref|XP_007868023.1|  hypothetical protein GLOTRDRAFT_139859, part...  42.7    1.3  


 >emb|CED83616.1| WW domain [Xanthophyllomyces dendrorhous]
Length=836

 Score = 1653 bits (4280),  Expect = 0.0, Method: Compositional matrix adjust.
 Identities = 836/836 (100%), Positives = 836/836 (100%), Gaps = 0/836 (0%)

Query  1    MDDEDENLVDWGYDDDEEVKGTPSSHGHPASDNADGFDALVGEDTTSQPSKGPSLLSRIS  60
            MDDEDENLVDWGYDDDEEVKGTPSSHGHPASDNADGFDALVGEDTTSQPSKGPSLLSRIS
Sbjct  1    MDDEDENLVDWGYDDDEEVKGTPSSHGHPASDNADGFDALVGEDTTSQPSKGPSLLSRIS  60

Query  61   GNIQPVFSPTSKKSVQNETQNASNSVRSSPNSGRPTTHRQSNDPTTGDSEKASLIEAAPS  120
            GNIQPVFSPTSKKSVQNETQNASNSVRSSPNSGRPTTHRQSNDPTTGDSEKASLIEAAPS
Sbjct  61   GNIQPVFSPTSKKSVQNETQNASNSVRSSPNSGRPTTHRQSNDPTTGDSEKASLIEAAPS  120

Query  121  QSAPSTSYPASSDPFSTTDQRSSSGHEELPPKISSNRQSTADSVRLKNSRPVDSHVPDAF  180
            QSAPSTSYPASSDPFSTTDQRSSSGHEELPPKISSNRQSTADSVRLKNSRPVDSHVPDAF
Sbjct  121  QSAPSTSYPASSDPFSTTDQRSSSGHEELPPKISSNRQSTADSVRLKNSRPVDSHVPDAF  180

Query  181  STQRRPPPPPLPRSQHSADEATETETTRPSTVTKEGPLSLDGLSAEWEIRTSRTSQGAKY  240
            STQRRPPPPPLPRSQHSADEATETETTRPSTVTKEGPLSLDGLSAEWEIRTSRTSQGAKY
Sbjct  181  STQRRPPPPPLPRSQHSADEATETETTRPSTVTKEGPLSLDGLSAEWEIRTSRTSQGAKY  240

Query  241  YFNKRTKQSSWERPEGTTATGSNADLNERDRHGSNKSDPTRSDRADTGPDRRRSGDWDRN  300
            YFNKRTKQSSWERPEGTTATGSNADLNERDRHGSNKSDPTRSDRADTGPDRRRSGDWDRN
Sbjct  241  YFNKRTKQSSWERPEGTTATGSNADLNERDRHGSNKSDPTRSDRADTGPDRRRSGDWDRN  300

Query  301  AASSRLTNYGSRSEDDRRRNRAEGWDLERNRHAGREGRSDISMNERRDRQAAIETAYKRP  360
            AASSRLTNYGSRSEDDRRRNRAEGWDLERNRHAGREGRSDISMNERRDRQAAIETAYKRP
Sbjct  301  AASSRLTNYGSRSEDDRRRNRAEGWDLERNRHAGREGRSDISMNERRDRQAAIETAYKRP  360

Query  361  SGLSSRSASNSFQEAHDWSTPGSRNGQSPSNHRRSSPAPGAAASGERPPSERTSQPTGDR  420
            SGLSSRSASNSFQEAHDWSTPGSRNGQSPSNHRRSSPAPGAAASGERPPSERTSQPTGDR
Sbjct  361  SGLSSRSASNSFQEAHDWSTPGSRNGQSPSNHRRSSPAPGAAASGERPPSERTSQPTGDR  420

Query  421  SLDLYRDKSRDKPPFPSTATTDERSNTLPARGEPIPSRDPVSGSEKRPQPVVPSGPRNIT  480
            SLDLYRDKSRDKPPFPSTATTDERSNTLPARGEPIPSRDPVSGSEKRPQPVVPSGPRNIT
Sbjct  421  SLDLYRDKSRDKPPFPSTATTDERSNTLPARGEPIPSRDPVSGSEKRPQPVVPSGPRNIT  480

Query  481  LDGPVGGEPYGRRRAPPVAGVNAVSAQGSTMRKWGPAASSVDERKQTTETADVSARENDD  540
            LDGPVGGEPYGRRRAPPVAGVNAVSAQGSTMRKWGPAASSVDERKQTTETADVSARENDD
Sbjct  481  LDGPVGGEPYGRRRAPPVAGVNAVSAQGSTMRKWGPAASSVDERKQTTETADVSARENDD  540

Query  541  KRREEDEAERERTRERERQRQRESDEKKRLEEERKREKNIARSRDKPREVVPAPSPAYDP  600
            KRREEDEAERERTRERERQRQRESDEKKRLEEERKREKNIARSRDKPREVVPAPSPAYDP
Sbjct  541  KRREEDEAERERTRERERQRQRESDEKKRLEEERKREKNIARSRDKPREVVPAPSPAYDP  600

Query  601  TAPFELSEEEKALILAKRQRAAQEEHARVIQKRDDHLPDGVQKTGEQATRDAYSRRRQPP  660
            TAPFELSEEEKALILAKRQRAAQEEHARVIQKRDDHLPDGVQKTGEQATRDAYSRRRQPP
Sbjct  601  TAPFELSEEEKALILAKRQRAAQEEHARVIQKRDDHLPDGVQKTGEQATRDAYSRRRQPP  660

Query  661  ETTVTSSYGHEQTSTPVRPTRDGAVRVISSVANSTGARPNMGPSIDLVDLTSPSPPRAVD  720
            ETTVTSSYGHEQTSTPVRPTRDGAVRVISSVANSTGARPNMGPSIDLVDLTSPSPPRAVD
Sbjct  661  ETTVTSSYGHEQTSTPVRPTRDGAVRVISSVANSTGARPNMGPSIDLVDLTSPSPPRAVD  720

Query  721  VESAVPSKRGHDALEPPQSSTQSREKNGNEDGDGDKVKDRVEEPRTNRARHQGIGPDDVN  780
            VESAVPSKRGHDALEPPQSSTQSREKNGNEDGDGDKVKDRVEEPRTNRARHQGIGPDDVN
Sbjct  721  VESAVPSKRGHDALEPPQSSTQSREKNGNEDGDGDKVKDRVEEPRTNRARHQGIGPDDVN  780

Query  781  DGQSRPVVPGWGPGSRRVGRGGIPERPPPYGLPRGGGRGGFGRGGRGDAWSGARSR  836
            DGQSRPVVPGWGPGSRRVGRGGIPERPPPYGLPRGGGRGGFGRGGRGDAWSGARSR
Sbjct  781  DGQSRPVVPGWGPGSRRVGRGGIPERPPPYGLPRGGGRGGFGRGGRGDAWSGARSR  836


>ref|XP_001884802.1| predicted protein [Laccaria bicolor S238N-H82]
 gb|EDR04630.1| predicted protein [Laccaria bicolor S238N-H82]
Length=1372

 Score = 47.0 bits (110),  Expect = 0.11, Method: Compositional matrix adjust.
 Identities = 26/68 (38%), Positives = 38/68 (56%), Gaps = 7/68 (10%)

Query  223  LSAEWEIRTSRTSQGAKYYFNKRTKQSSWERP------EGTTATGSNADLNERDRHGSNK  276
            L +EWE+R  R S G  YY+N  T QS+W RP       GT+A+  + D+ E  +H S+ 
Sbjct  207  LPSEWEVRHPR-SGGGVYYYNSETHQSTWTRPPSLRATRGTSASQPDFDITEASKHPSSS  265

Query  277  SDPTRSDR  284
            +  +  DR
Sbjct  266  NPLSYEDR  273


>ref|XP_001838984.2| hypothetical protein CC1G_05537 [Coprinopsis cinerea okayama7#130]
 gb|EAU82915.2| hypothetical protein CC1G_05537 [Coprinopsis cinerea okayama7#130]
Length=1166

 Score = 45.4 bits (106),  Expect = 0.29, Method: Compositional matrix adjust.
 Identities = 41/122 (34%), Positives = 52/122 (43%), Gaps = 22/122 (18%)

Query  159  STADSVRLKN---SRPVDSHVPDAFSTQRRPPPPPLPRSQHSADEATETETTRPSTVTKE  215
            S  DS R KN   SRP   H P A  T   PP P +         AT   T+  S  ++ 
Sbjct  82   SREDSTRPKNGSPSRPQQIHQPPARLTHALPPKPTV--QDVPIAPATAKATSMKSVSSRA  139

Query  216  GPLSLDG---------------LSAEWEIRTSRTSQGAKYYFNKRTKQSSWERPEGTTAT  260
             P   +G               L  +WEIR SR  +G  YY+N RT++S W RP  +   
Sbjct  140  APKKANGAPTTKNPSSTDLDRDLPPDWEIRYSR--KGEPYYWNTRTRESRWTRPAHSVGD  197

Query  261  GS  262
            GS
Sbjct  198  GS  199


>ref|XP_007868023.1| hypothetical protein GLOTRDRAFT_139859, partial [Gloeophyllum 
trabeum ATCC 11539]
 gb|EPQ53731.1| hypothetical protein GLOTRDRAFT_139859, partial [Gloeophyllum 
trabeum ATCC 11539]
Length=353

 Score = 42.7 bits (99),  Expect = 1.3, Method: Compositional matrix adjust.
 Identities = 17/38 (45%), Positives = 25/38 (66%), Gaps = 0/38 (0%)

Query  219  SLDGLSAEWEIRTSRTSQGAKYYFNKRTKQSSWERPEG  256
            S D L  +WE+RT R      YY+N +T++S+W+RP G
Sbjct  16   SDDQLPPDWEMRTPRGGGSNPYYYNTKTQESTWDRPSG  53


Lambda      K        H        a         alpha
   0.306    0.125    0.362    0.792     4.96 

Gapped
Lambda      K        H        a         alpha    sigma
   0.267   0.0410    0.140     1.90     42.6     43.6 

Effective search space used: 9887908777281


  Database: nr
    Posted date:  Sep 23, 2015 12:05 AM
  Number of letters in database: 26,053,659,533
  Number of sequences in database:  71,551,133


Matrix: BLOSUM62
Gap Penalties: Existence: 11, Extension: 1
Neighboring words threshold: 11
Window for multiple hits: 40
```
